# Complex Conjugated certificateless-based signcryption with differential integrated factor for secured message communication in mobile network

**DOI:** 10.1371/journal.pone.0186207

**Published:** 2017-10-17

**Authors:** Sumithra Alagarsamy, S. P. Rajagopalan

**Affiliations:** 1 Part Time Research Scholar, Department of Information and Communication Engineering, Anna University, Guindy, Chennai, Tamilnadu, India; 2 Department of Computer Science and Engineering, G. K. M. College of Engineering and Technology, Perungalathur, Chennai, Tamilnadu, India; school of science, Beijing University of Chemical Technology, CHINA

## Abstract

Certificateless-based signcryption overcomes inherent shortcomings in traditional Public Key Infrastructure (PKI) and Key Escrow problem. It imparts efficient methods to design PKIs with public verifiability and cipher text authenticity with minimum dependency. As a classic primitive in public key cryptography, signcryption performs validity of cipher text without decryption by combining authentication, confidentiality, public verifiability and cipher text authenticity much more efficiently than the traditional approach. In this paper, we first define a security model for certificateless-based signcryption called, Complex Conjugate Differential Integrated Factor (CC-DIF) scheme by introducing complex conjugates through introduction of the security parameter and improving secured message distribution rate. However, both partial private key and secret value changes with respect to time. To overcome this weakness, a new certificateless-based signcryption scheme is proposed by setting the private key through Differential (Diff) Equation using an Integration Factor (DiffEIF), minimizing computational cost and communication overhead. The scheme is therefore said to be proven secure (i.e. improving the secured message distributing rate) against certificateless access control and signcryption-based scheme. In addition, compared with the three other existing schemes, the CC-DIF scheme has the least computational cost and communication overhead for secured message communication in mobile network.

## 1. Introduction

With therapid progress in mobile communication networks, certificateless-based signcryption are under research and development stage. An access control scheme was designed in [1] using ceritificateless signcryption, achieving confidentiality, integrity and authentication. Yet another novel light weight ECC based key distribution model as designed in [2] measured the energy consumption and execution time with respect to each operation for resource constrained devices.

One of the emerging network paradigmsthat achieve interactions between pervasive models through heterogeneous networks is the Internet of Things (IoT).A certificateless offline signcryption model was designed in [3] for resource constrained devices. However, correct decisions regarding secure communications remained unaddressed. To solve this issue, in [4], a Certificateless Multi Recipient Encryption Scheme (CL-MRES) was designed. The scheme collected data from hundreds of small sensors via dual channel strategy. Another formal security model for certificateless signcryption scheme was presented in [5].

In recent years, privacy preservation and secured communication are gaining popularity due to the advent of mobile network. A heterogeneous signcryption scheme was presented in [6] with no restriction on system parameters and master keys. Yet another certificateless signcryption scheme was structured in [7] using bilinear pairings. The scheme was proved to be confidential with the application of modified decisional bilinear Diffie–Hellman (M-DBDH).Using bilinear pairings though security was said to be achieved but at the cost of computation. To address this issue, a cryptanalysis mechanism in addition to certificateless signcryption without pairing was demonstrated in [8].Yet another, computational review of identity based signcryption schemes was provided in [9].

Internet of Things (IoT) deals with the interconnection of devices, comprising of smart objects, embedded computing devices and so on. In [10], elliptic curve-based signcryption scheme, from the standardized signature was proposed ensuring not only security but also minimizing computation and communication overhead. However, to increase security without pairing operation, cryptographic hash function was designed in [11]. However, the scheme was proven to have certain security weakness. To address this issue, computational Diffie-Hellman assumption was extended [12] and therefore was found to be secured with lesser public parameter size.

The concept of aggregate signcryption was first introduced in [13]. The aggregation process of these schemes not only reduced the amount of exchanged information but was also proven to be specifically useful in low-bandwidth communication networks and computationally- restricted environments. However, it suffered from the key escrow problem. To address this issue a suitable security model for aggregate signcryption in the certificateless setting was designed in [14].

A hybrid signcryption scheme in certificateless setting was designed in [15] by extending the concept of signcryption tag-KEM to the certificateless setting. The scheme was not only proven to be more secured but was also found to the most cost efficient model. An identity-based signcryption scheme was designed in [16] to address the security weakness in the above said methods. A client-based programming called java script was used in [17] to minimize the workload of server to achieve system security.

Based on the aforementioned methods and technique, the main contributions of this paper are summarized as follows:

- AComplex Conjugated Certificateless Signcryption scheme, called, Complex Conjugate Differential Integrated Factor (CC-DIF) which improves secured message distribution rate on the basis of master secret key generation using complex conjugated factor is presented. With the complex conjugated factor, the polynomial having complex zero, as the conjugate of that zero is also its one of the root, message distributing rate is said to be secured.The proposed scheme considers two security parameters for obtaining complex conjugated form.
- The memory consumption is reduced by extracting partial private key based on the arbitrary key bits considering both the random and modulus operand for each user ‘*U*’ with user identity ‘*ID*’ in the network. With the extraction of partial private key and arbitrary key bits generated in CC-DIF scheme, storage process is said to be reduced. This is because as only partial private keys are extracted and arbitrary key bits are generated for each user that varies for different session.
- By efficient generation of random user key, computation cost is identified to be reduced using random value whenever a new user enter into the network. In this way, the genuinity of the user is ensured. The computation cost involved in CC-DIF scheme is said to be genuine because of the random generation of user key which is said to be changed each and every time upon entering of a new user.
- Finally, the proposed scheme reduces communication overhead between users by applying differential equation and an integration factor.

The rest of the paper is organized as follows. The CC-DIF scheme is introduced in Section 2 with the aid of the flow diagram and algorithmic description. The performance is evaluated in Section 3. Simulation results are discussed in Section 4. Finally, the concluding remarks are summarized in Section 5.

## 2. Complex Conjugate Differential Integrated Factor

**Fig 1:**
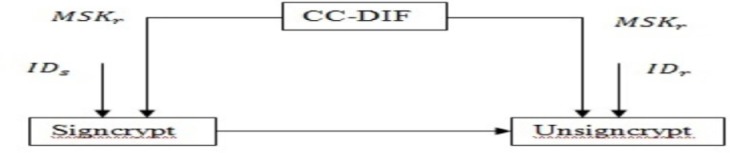
Complex Conjugated certificateless signcryption scheme.

The algorithm for Complex Conjugate Differential Integrated Factor scheme (CC-DIF) = (Setup,KeyGenration, Signcrypt, Unsigncrypt) consists of five algorithms, namely, Complex Conjugated Master Secret Key Generation for Setup, Arbitrary Key Partial Private Key Extraction, Random User Key Generation, Private Key using Differential Equated Integration Factor for KeyGenration, Signcrypt/Unsigncrypt. Fig 1 shows the Complex Conjugated Certificateless Signcryption scheme.

As shown in Fig 1, the Certificateless Signcryption scheme is performed through Differential and Integrated Factor (DiffEIF) with the aid of the master key ‘*MSK*’ generated by asender ‘*s*’ for a receiver ‘*r*’. In a similar manner, unsigncryption is performed with the aid of master key ‘*MSK*’ for the receiver ‘*r*’ send by a sender ‘*s*’ respectively.

In this section, we extend the concept of certificateless-based signcryption with security features using Complex Conjugate Differential Integrated Factor (CC-DIF). The flow diagram for CC-DIF scheme is as given below.

**Fig 2:**
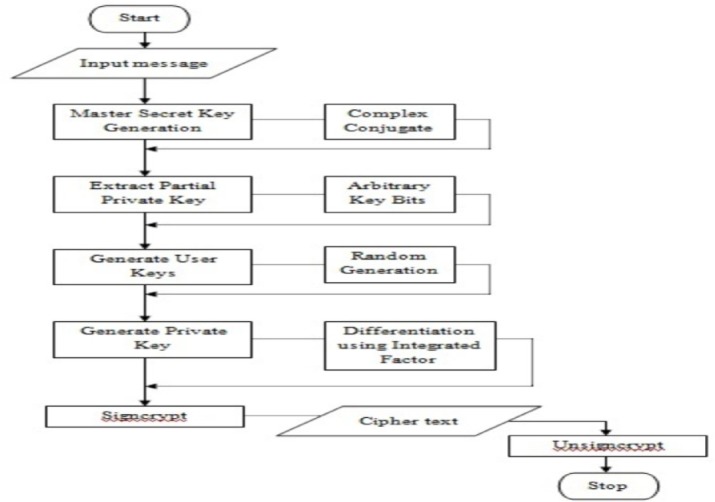
Flow diagram of CC-DIF.

As shown in the Fig 2, the Complex Conjugated certificateless signcryption scheme based on differentiation using an integration factor generates master secret key using complex conjugated factor. The advantage of applying complex conjugated factor is that if the degree of a master key is odd, it must have at least one real root, and hence said to be more secured. With this master secret key obtained, partial private key are extracted via arbitrary key bits. The purpose of using arbitrary key bits is that it is said to be highly unpredictable. At the same time, as it is only the partial private key, the key values need to be the same all the time and hence said to be more secured.

With the partial private keys extracted, user keys are generated with the aid of random generation model and the private keys are extracted via differentiation using an integration factor (DiffEIF). Hence, with the random generation model, the drudgery of saving the user keys are said to be reduced highly, reducing the computation cost and overhead. Followed by this, signcryption is performed by the sender to obtain cipher text for corresponding message that has to be sent to the intended recipient. With the obtained cipher text, unsigncryption is performed on the part of the receiver to obtain original message as sent by the sender. With imperfect matching, an error message is reported in order to continue to process with the other set of sender for the intended recipients or receivers. This process is continued until all the senders are processed with.

### 2.1 Master Secret Key Generation using Complex Conjugated form

The setup phase in CC-DIF scheme provides security parameters ‘*I*^*k*^ ∈ *λ*_1_,*λ*_2_’ as input and obtains Master Secret Key ‘*MSK*’ using Complex Conjugate form. In addition to the system parameters, the setup phase includes Master Public Key ‘*MPK*’, message space ‘*ms*’ and cipher text space ‘*cs*’ respectively and is as given below.

params→(MPK,ms,cs) (1)

The Complex Conjugate form for generating ‘*MSK*’ is as given below. Let us consider ‘*I*^*k*^ ∈ *λ*_1_,*λ*_2_’, where ‘*λ*_1_ ∈ *e*^*λ*1*t*^, *λ*_1_ ∈ *e*^*λ*2*t*^’, then the Complex Conjugate of ‘*λ*_1_,*λ*_2_’, with the generation of ‘*MSK*’ is as given below

$$MSK=c_{1}e^{\lambda 1t}+c_{1}e^{\lambda 2t}$$ (2)

From (2), the value of Master Secret Key ‘*MSK*’ is obtained using the complex conjugate form of the security parameter ‘*λ*_1_’ and the security parameter ‘*λ*_2_’ respectively at time ‘*t*’. The algorithmic representation for master secret key generation (algorithm 1) is as given below and the details in implementing the algorithm are given with case examples provided in S1 Case File.

**Algorithm 1:** **Complex Conjugated Master Secret Key Generation**

**Input**: Users ‘*U*’, Security Parameters ‘*λ*_1_,*λ*_2_’

**Output**: System Parameters ‘*params*’, Master Secret Key ‘*MSK*’

1: **Begin**

2:         **For** all users ‘*U*’ with security parameters ‘*λ*_1_,*λ*_2_’

3:                 Generate System Parameters using (1)

4:                 Generate Complex Conjugate using (2)

5:         **End for**

6: **End**

As given above, with security parameters as input, for all ‘*n*’ users, master secret key and system parameters are generated as output. The system parameters includemaster public key, space for message and cipher text. On the other hand, the output master secret key is generated with the aid of complex conjugation, where linearly independent terms are obtained using ‘*e*^*λ*1*t*^’ and ‘*e*^*λ*2*t*^’.

### 2.2 Arbitrary Partial Private Key Extraction

Once the Master Secret Key ‘*MSK*’ is generated using the Complex Conjugated form, Partial Private Key ‘*D*_*ID*_’ is extracted using Arbitrary Key Bits. The Arbitrary Key Bits include a random number for each user ‘*U*’ with user identity ‘*ID*’ in addition to the modulus operand of ‘*ith*’ user. The Arbitrary Key Bits ‘*D*_*ID*_’ generated is as given below in (3).

$$D_{ID}\to \left(Rand\;\left(U\left(ID\right)\;mod\;i\right)\right)$$ (3)

The algorithm for Arbitrary Partial Private Key Extraction is as given below (algorithm 2). The algorithm in S2 Appendix is the implementation of the partial private key extraction using arbitrary bits serial bisecting method for secure message communication in mobile network.

**Algorithm 2:** **Arbitrary Partial Private Key Extraction**

**Input**: Users ‘*U*’, System Parameters ‘*params*’, Master Secret Key ‘*MSK*’, User Identity ‘*ID*’

**Output**: Partial Private Key ‘*D*_*ID*_’

1: **Begin**

2:         **For** each user ‘*U*’ with User Identity ‘*ID*’

3:                 **If** User Identity ‘*ID* = 1’

4:                         Return Partial Private Key ‘*D*_*ID*_’ using (3)

5:                 **End if**

6:                 **If** User Identity ‘*ID* = 0’

7:                         Return abnormal users

8:                 **End if**

9:         **End for**

10: **End**

As shown above, the algorithm for Arbitrary Partial Private Key Extraction includes System Parameters ‘*params*’, Master Secret Key ‘*MSK*’, User Identity ‘*ID*’ with which the Partial Private Key is obtained. With the generation of Partial Private Key using Arbitrary Key, the drudgery of maintaining key for all user’s are relieved, as the key are not necessary to be stored in memory, with each user provided with different Partial Private Key at different time. Hence, the memory consumption involved during message communication is said to be reduced.

### 2.3 Random User Key Generation

The third step involved in the CC-DIF scheme is the generation of user keys. In this step, the User Identity ‘*ID*’ and Master Public Key ‘*MPK*’ are taken as input for which the corresponding secrete value ‘*x*_*ID*_’ and public key ‘*PK*_*ID*_’ are obtained as output. The random user key generation is as given below in (4) and (5).

$$x_{ID}\to Rand\;(ID)$$ (4)

$${PK}_{ID}\to Rand\;(MPK)$$ (5)

The algorithm for Random User Key Generation is as given below (algorithm 3).

**Algorithm 3:** **Random User Key Generation**

**Input**: Users ‘*U*’, User Identity ‘*ID*’, Master Public Key ‘*MPK*’

**Output**: sec1: **Begin**

2:         **For** each user ‘*U*’ with User Identity ‘*ID*’ and Master Public Key ‘*MPK*’

3:                 Obtain secrete value ‘*x*_*ID*_’ using (4)

4:                 Obtain public key ‘*PK*_*ID*_’ using (5)

5:         **End for**

6: **End** ret value ‘*x*_*ID*_’, public key ‘*PK*_*ID*_’

The input for the Random User Key Generation algorithm comprises of the Users ‘*U*’, User Identity ‘*ID*’, Master Public Key ‘*MPK*’ with the output being secret value ‘*x*_*ID*_’, public key ‘*PK*_*ID*_’. The output is arrived at with the aid of the random number of user ID and Master Public Key resulting in secret value ‘*x*_*ID*_’ and public key ‘*PK*_*ID*_’ respectively.

### 2.4 Differential Equated Private Key using an Integration Factor

In this step, private keys are set using Differential Equation and an Integration Factor (DiffEIF). With the secret value ‘*x*_*ID*_’ and public key ‘*D*_*ID*_’ as input, the first order differential equation is as given below.

$$\frac{dy}{dx}+D_{ID}y+x_{ID}y=P$$ (6)

From (6), ‘*D*_*ID*_’, ‘*x*_*ID*_’ and ‘*P*’ are functions involving ‘*x*’ only. Let us further multiply both sides of the differential equation by an integrating factor ‘*I*’ which is defined as below in (7).

$$I=e^{\int D+x\;dx}$$ (7)

Multiplying the original differential equation by ‘*I*’ we get

$$\int I(\frac{dy}{dx})+I(D_{ID}y)+I(x_{ID}y)=\int IP$$ (8)

$$\int (I\;\frac{dy}{dx}+ID_{ID}+Ix_{ID})\;dx=\int IP\;dx$$ (9)

$$2Iy=\int IP\;dx,\;since\;\frac{d}{dx}\;2Iy=I\;\frac{dy}{dx}+ID_{ID}+Ix_{ID}$$ (10)

$$S_{ID}=2Iy=\int IP\;dx$$ (11)

From (8), (9), (10) and (11), the full private key ‘*S*_*ID*_’ is obtained. The algorithm for Differential Equated Private Key using an Integration Factor is as given below (algorithm 4).

**Algorithm 4.** **Private Key using Differential Equated Integration Factor**

**Input**: Users ‘*U*’, User Identity ‘*ID*’, secret value ‘*x*_*ID*_’, public key ‘*D*_*ID*_’

**Output**: Full Private Key ‘*S*_*ID*_’

1: **Begin**

2:         **For** each user ‘*U*’ with User Identity ‘*ID*’

3:                 Obtain first order differential equation using (6)

4:                 Obtain an integrating factor ‘*I*’ using (7)

5:                 Multiplyoriginal differential equation by ‘*I*’ using (8)

6:                 Obtain Full Private Key ‘*S*_*ID*_’ using (11)

7:         **End for**

8: **End**

The Private Key using Differential Equated Integration Factor algorithm takes as input Users ‘*U*’, User Identity ‘*ID*’, secret value ‘*x*_*ID*_’, public key ‘*D*_*ID*_’ and returns Full Private Key ‘*S*_*ID*_’ as output. This is obtained using the Differential Equation form for secret value and public key to which an integration factor is applied to obtain Full Private Key ‘*S*_*ID*_’ respectively.

### 2.5 Signcrypt

Upon successful generation of the keys, signcrypt is performed by the sender, whereas unsigncrypt is performed on the receiving end by the receiver. In addition to the signcrypt and unsigncrypt function, a function ‘*f*’ stating to perform signcrypt or unsigncrypt is also provided in algorithm 5. Here, the function ‘*f*’, includes ‘(0,1)’, with ‘0’ symbolizing the signcrypt whereas ‘1’ symbolizing the unsigncrypt.

The input to the signcrypt function includes, the system parameters, message ‘*M*’, Senders Full Private Key ‘$S_{{ID}_{s}}$’, Senders Identity ‘*ID*_*s*_’, Receivers Public Key ‘${PK}_{{ID}_{r}}$’, Receivers Identity ‘*ID*_*r*_’. The signcrypt function is performed by XOR-ing the message ‘*M*’, senders full private key ‘$S_{{ID}_{s}}$’ and receivers public key ‘${PK}_{{ID}_{r}}$’ respectively ‘$M\;⨁S_{{ID}_{s}}\;⊕\;{PK}_{{ID}_{r}}$’. The signcrypt function (12) is as given below.

$$\sigma \to Signcrypt\;\left((M\;⊕\;S_{{ID}_{s}}⊕\;{PK}_{{ID}_{r}})\right)$$ (12)

The output to the above function ‘*Signcrypt*()’ is the cipher text ‘*σ*’. On the other hand, at the receiving end, the receiver performs the unsigncrypt function with the input as system parameters ‘*params*’, cipher text ‘*σ*’, Receivers Public Key ‘${PK}_{{ID}_{r}}$’, Senders Public Key ‘${PK}_{{ID}_{s}}$’, Receivers Full Private Key ‘$S_{{ID}_{r}}$’, Receivers Identity ‘*ID*_*r*_’. The unsigncrypt function (13) is as given below.

$$M\to Unsigncrypt\;(params,\;\sigma ,{ID}_{s},\;{PK}_{{ID}_{s}},S_{{ID}_{r}},\;{ID}_{r},\;{PK}_{{ID}_{r}})$$ (13)

The output to the above function ‘*Unsigncrypt*()’ is the original text message ‘*M*’. The algorithm for signcrypt and unsigncrypt function is as given below (algorithm 5).The program in S3 Appendix will give the details of the implementation in the signcryption and unsigncryption in order to perform secure message communication between users.

**Algorithm 5.** **Signcrypt and Unsigncrypt.**

**Input**: plain text message ‘*M*’, system parameters ‘*params*’, function ‘*f*’, Senders Full Private Key ‘$S_{{ID}_{s}}$’, Senders Identity ‘*ID*_*s*_’, Senders Public Key ‘${PK}_{{ID}_{s}}$’, Receivers Identity ‘*ID*_*r*_’, Receivers Public Key ‘${PK}_{{ID}_{r}}$’, Senders Public Key ‘${PK}_{{ID}_{s}}$’, Receivers Full Private Key ‘$S_{{ID}_{r}}$’, Receivers Identity ‘*ID*_*r*_’

**Output**: Secured message communication

1: **Begin**

2:         **For** each user ‘*U*’ with User Identity ‘*ID*’ and function ‘*f*’

3:                 **If** function ‘*f* = 0’

4:                         Obtain plain text message ‘*M*’ with Senders Full Private Key ‘$S_{{ID}_{s}}$’, Senders Identity ‘*ID*_*s*_’ and Senders Public Key ‘${PK}_{{ID}_{s}}$’

5:                         Obtain Receivers Identity ‘*ID*_*r*_’ and Receivers Public Key ‘${PK}_{{ID}_{r}}$’

6:                         Perform signcrypt using (12)

7:                         Obtain the cipher text ‘*σ*’

8:                 **End if**

9:                 **If** function ‘*f* = 1’

10:                         Obtain cipher text ‘*σ*’ with Receivers Full Private Key ‘$S_{{ID}_{r}}$’, Receivers identity ‘*ID*_*r*_’, Receivers Public Key ‘${PK}_{{ID}_{r}}$’

11:                 Obtain Senders identity ‘*ID*_*s*_’ Senders Public Key ‘${PK}_{{ID}_{s}}$’

12:                 Perform unsigncrypt using (13)

13:                 Obtain original message ‘*M*’

14:         **End if**

15:     **End for**

16: **End**

As given in the above algorithm, with the function involving signcrypt, cipher text is generated by the sender for secured transmission of messages in mobile network. On the other hand, original message is generated by the receiver via unsigncrypt function. In this way, secured message communication in mobile network is said to be achieved.

## 3. Experimental setup

The scheme called Complex Conjugate Differential Integrated Factor for secured message communication in mobile network uses the NS-2 simulator with the network range of 1000*1000 m size. To conduct experimental work, Dynamic Source Routing (DSR) protocol is used as routing protocol for CC-DIF scheme.The CC-DIF scheme’s node moving speed or the communication between the user’s in mobile networking is about 2 to 25 m/s for each user with a simulation rate of 600 seconds to perform secured communication between users. Each simulation is carried out under a differentnumber of network nodes and the performance metrics areobtained by averaging over 10 simulation runs. The parametric values for performing experiments are shown in Table 1.

**Table 1:** Simulation parameter.

Parameter	Value
Simulator	NS-2.31
Number of nodes	50, 100, 150, 200, 250, 300, 350, 400, 450, 500
Network area	1000m * 1000m
Transmission range	250m
Number of messages	5, 10, 15, 20, 25, 30, 35, 40, 45, 50
Simulation period	600s
Node speed	2–25 m/s
Node pause time	0–300 seconds
Routing protocol	Dynamic Source Routing Protocol (DSR)
Number of runs	10

Experiment is conducted on factors such as message size, message sent, computation cost, memory consumption, communication overhead and secured message distribution rate for mobile network. The results of the metrics of CC-DIF scheme is compared against the existing methods such as Certificate Less Sign Cryption (CLSC) [1], Elliptic Curve Cryptography-based (ECC) Multimedia Internet KEYing (MIKEY) [2] and Certificateless online/offline signcryption (COOSC) scheme for IoT [3].

## 4. Discussion

To validate the efficiency and theoretical advantages of Complex Conjugate Differential Integrated Factor (CC-DIF) scheme with Certificate Less Sign Cryption (CLSC) [1], Elliptic Curve Cryptography-based (ECC) Multimedia Internet KEYing (MIKEY) [2] and Certificateless online/offline signcryption (COOSC) scheme for IoT [3], simulation results under NS2 are presented. The parameters of the CC-DIF scheme are chosen as provided in the experiment section with graph comparisons provided in S1 Graph File.

### 4.1 Computation cost

To better understand the effectiveness of the proposed CC-DIF scheme, with respect to computation cost, extensive experimental results are reported in Table 2. Computation cost is a measure of cost involving the time taken to perform key generation (i.e. partial private key extraction) with respect to the message size provided as input.

$$CC=Time(D_{ID})*Size(M)$$ (14)

**Table 2:** Tabulation for computation cost.

Message size (KB)	Computation Cost			
	CC-DIF	COOSC	ECC-based MIKEY	CLSC
15	16.8	18.6	23.7	28.9
30	19.6	23.2	27.6	32.8
45	29.1	31.3	35.1	40.1
60	39.4	41.3	45.3	50.2
75	42.14	45.90	50.6	55.3
90	48.52	50.32	53.8	58.6
105	49.32	52.14	55.4	60.3
120	55.16	57.32	61.3	66.1
135	59.10	60.23	64.9	69.8
150	61.32	64.13	66.6	71.2

From (14), the computation cost ‘*CC*’ is obtained using the time for partial key extraction ‘*Time*(*D*_*ID*_)’ and the message size ‘*Size*(*M*)’ respectively.

NS2 simulator investigates the computation cost by analyzing the result using table and graph values. Results are presented for different message sizes in the range of 15KB to 150KB. The results reported here confirm that with the increase in the message size, the computation cost also gets increased and found to be linear.

**Fig 3:**
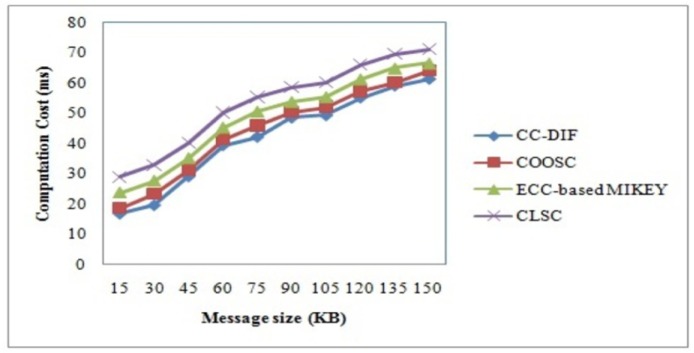
Computation cost of CC-DIF and three existing methods.

Fig 3 shows computation cost based on the message sizes sent by the sender to the intended recipients in mobile network. Our proposed CC-DIF scheme performs relatively well when compared to three other methods,Certificate Less Sign Cryption (CLSC) [1], Elliptic Curve Cryptography-based (ECC) Multimedia Internet KEYing (MIKEY) [2] and Certificateless online/offline signcryption (COOSC) scheme for IoT [3]. The computation cost is reduced in the CC-DIF scheme by applying random user key generation. With the aid of random user key generated for each user, the key values are not stored in memory. Therefore, the message is securely communicated to the intended recipient during each time, random user key generated different for each users. This in turn reduces computation cost involved during message communication in mobile network. By applying random user key generation scheme, efficient communication is said to take place where random ID and random value for master public key is generated for each user. This in turn reduces the computation cost using CC-DIF scheme by 8% compared to COOSC and 19% compared to ECC-based MIKEY. Moreover, the user does not have to fetch the key values as it is generated differently for different number of time with theaid of random keys that in turn helps in reducing the computation cost using CC-DIF scheme by 33% compared to CLSC.

### 4.2 Memory consumption

As listed in Table 3, CC-DIF scheme measures memory consumption with respect to message sizes in the range of 15KB to 150KB. It is measured in terms of Kilo Bytes (KB). The memory consumption for secured message communication in mobile network using CC-DIF scheme offers comparable values than the state-of-the-art methods.Memory consumption for key extraction is measured using the available and used memory in mobile network. The memory consumption is mathematically formulated as given below.

$$MC=M_{avail}-M_{used}$$ (15)

**Table 3:** Tabulation for memory consumption.

Message size (KB)	Memory consumption (KB)			
	CC-DIF	COOSC	ECC-based MIKEY	CLSC
15	113	129	136	148
30	125	135	143	155
45	148	152	166	178
60	185	200	208	220
75	212	225	235	235
90	228	235	247	247
105	235	248	260	260
120	256	265	280	280
135	270	284	296	296
150	292	205	312	312

From (15) the memory consumption ‘*MC*’, is obtained using the available memory ‘*M*_*avail*_’, and the used memory ‘*M*_*used*_’, respectively.

The targeting results of memory consumption using CC-DIF scheme with three state-of-the-art methods [1], [2] and [3] in Table 3 presented for comparison based on the message sizes in mobile network. Higher the message size the rate of memory consumption is also said to be increased.

**Fig 4:**
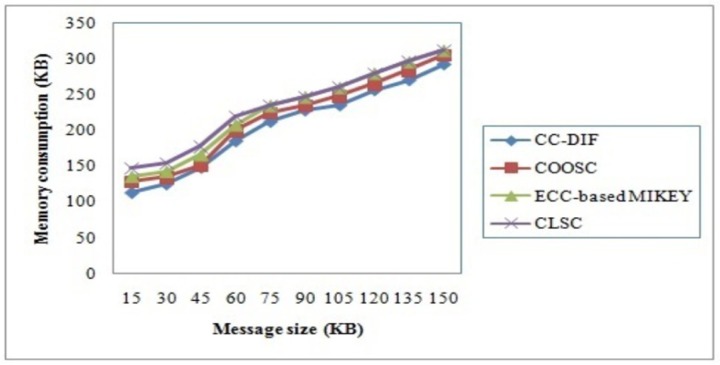
Memory consumption of CC-DIF and three existing methods.

Fig 4 presents the variation of memory consumption with respect to varied message sizes for mobile network. All the results provided in Fig 4 confirm that the proposed CC-DIF scheme significantly outperforms the other three methods, CLSC [1], ECC-based MIKEY [2] and COOSC [3]. The memory consumption though increases with the increase in the message size, but found to be comparatively lesser using CC-DIF scheme with the aid of Arbitrary Key Partial Private Key Extraction algorithm. In CC-DIF scheme, Partial Private Key for each sender is generated using Arbitrary Key Bits in addition to the modulus operand of ‘*ith*’ user. This not only generates different partial private key for each sender in an arbitrary manner but also reduces the Key Bits with the aid of the modulus operand. Hence, the memory consumption using the CC-DIF scheme is said to be reduced considerably by 6% compared to COOSC and 12% compared to ECC-based MIKEY. Furthermore, only authenticated users are provided with the partial private key where the CC-DIF scheme checks for the user identity with identity value of ‘1’ being generated with the partial private key whereas with the identity value of ‘0’ being considered as abnormal users. Hence, the memory consumption is said to be reduced in an efficient manner using CC-DIF scheme by 15% compared to CLSC.

### 4.3 Communication overhead

To better understand the effectiveness of the CC-DIF scheme, extensive experimental results are reported in Table 4 with respect to the number of messages being sent by different senders to different receivers. The communication overhead is defined as the total number of messages to be transferred from the sender to the receiver. It includes, the key generation time, performing signcryption and the unsigncryption.

$$CO=\sum_{i=1}^{n}M_{i}*(Time\;(Full\;Private\;Key\;‘S_{ID}’\;)+\;Time\;(Signcrypt)+\;Time\;(Unsigncrypt))$$ (16)

**Table 4:** Tabulation for communication overhead.

Number of message sent	Communication overhead (ms)			
	CC-DIF	COOSC	ECC-based MIKEY	CLSC
5	5.25	6.13	7.85	16.3
10	6.16	9.32	11.34	18.6
15	8.35	12.57	18.35	20.8
20	10.23	16.89	21.43	26.3
25	16.68	18.90	25.90	30.2
30	18.35	24.13	28.98	35.6
35	24.32	29.16	35.13	38.1
40	32.14	35.11	42.32	41.2
45	38.98	41.32	47.14	42.5
50	45.32	51.32	55.32	45.8

The targeting results of communication overhead using CC-DIF scheme with three state-of-the-art methods [1], [2] and [3] in Table 4 presented for comparison based on the number of messages being sent in mobile network.

**Fig 5:**
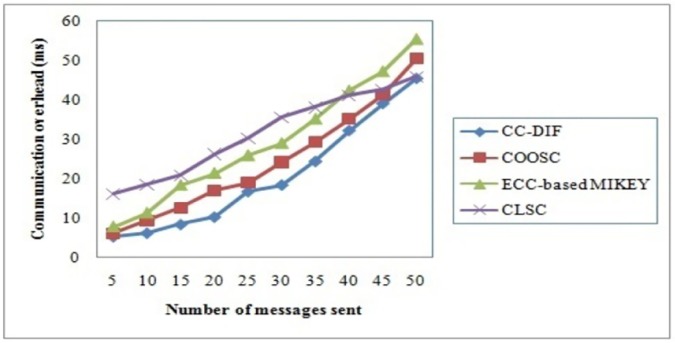
Communication overhead of CC-DIF and three existing methods.

Fig 5 presents thecommunication overhead versus different number of messages being sent using three methods CLSC [1], ECC-based MIKEY [2] and COOSC [3]. This figure shows that CC-DIF scheme causes lesser number of routing overheads when compared to three methods namely, CLSC [1], ECC-based MIKEY and COOSC [3] mainly becauseof Differential Equation and an Integration Factor (DiffEIF) are considered. To explore the bestperformance delivery path, CC-DIF scheme uses the Private Key using Differential Equated Integration Factoralgorithm, by initially generating a differential equation form to which an integration factor is applied. In CLSC [1], ECC-based MIKEY [2] and COOSC [3], communication is performed through access control scheme and key distribution mechanism for message communication. Hence, if the current route path fails, the three methods [1], [2] and [3] have to re-establish a path or again perform access control or key generation and distribution for each sender receiver pair from the scrap. This in turn consumes much more communicating overhead. However, in CC-Dif scheme, private keys are set using first order differential equation and as a result, the communication overhead using CC-DIF scheme is found to be comparatively lesser by 27% compared to COOSC, 50% compared to ECC-based MIKEY. In addition, by obtaining an integrating factor only for the differential integrated for each user with the authenticated user identity, the CC-DIF scheme is considerably said to be reduced by 78% compared to CLSC respectively. Therefore, our Private Key using Differential Equated Integration Factor is more suitable than compared to the state-of-the-art works.

### 4.4 Secured message distribution rate

Secured Message distributing rate is the ratio of message received at the receiving end ‘*M*_*r*_’ to the messages sent from the sender side ‘*M*_*s*_’. The secured message distributing rate is measured in terms of percentage (%) and is mathematically formulated as given below.

$$\;SMDR=\left(\frac{M_{r}}{M_{s}}\right)\;*100$$ (17)

Table 5 given below shows the results of secured message distributing rate for CC-DIF scheme and compared with three different methods.

**Table 5:** Tabulation for secured message distributing rate.

Number of messages sent	Secured message distribution rate (%)			
	CC-DIF	COOSC	ECC-based MIKEY	CLSC
5	78.23	75.14	70.41	65.36
10	84.15	80.23	73.20	68.15
15	90.25	83.14	75.15	70.10
20	91.34	85.14	77.57	72.52
25	92.85	89.32	79.73	74.68
30	93.14	90.23	83.70	78.65
35	94.13	92.15	85.20	80.10
40	95.14	93.13	82.55	82.45
45	96.11	94.14	89.25	84.65
50	96.28	95.13	91.75	86.65

**Fig 6:**
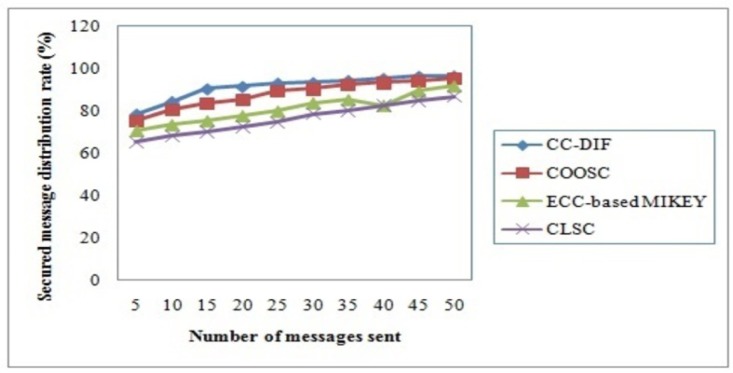
Secured message distribution rate of CC-DIF and three existing methods.

Fig 6 shows the secured message distribution rate of CC-DIF and comparison is made with three existing methods with respect to the different number of messages being sent. As shown in the figure, the secured message distribution rate increases with the increase in the number of messages being sent using all the methods. But it is observed to be comparatively better using the CC-DIF scheme. This is because of the application of Master Secret Key Generation using Complex Conjugated form. With the Complex Conjugated form, the master secret key generated is observed to be not only secured but also improving the message distribution rate. By applying CC-DIF scheme, the message distribution rate is found to be improved by 4% compared to CLSC, 11% compared to ECC-based MIKEY and 16% compared to COOSC.

## 5. Conclusion

Certificateless-based Signcryption is an indispensable operation in mobile networksto guarantee secured message communication, reduce computation overhead and computation cost. Inthis paper, we present a scheme to investigate complex conjugate differential integrated factor to ensure secured message communication between users with reduced memory consumption between users in mobile networks. The result indicates thatthe Complex Conjugates with DiffEIF have significantroles in evaluating secret key and partial private key. Based onthe developed formulas, the interaction between the users (i.e. senders and receivers) are studied which provide improved rate of message distribution and therefore found to be more secured. The proposed mathematical formulae provided as well asthe algorithms developed provides a manner for efficient signcrypt and unsigncrypt. With the emerging mobile technologies, certificateless scheme will be an important feature in the future-generation mobile networks. Furthermore, in thispaper, we focus on the user authenticity ensuring proper user identity proceeded with the process of returning private partial key and others treated as abnormal users. This kind of wireless networks is significantlydifferent from multihop networks, e.g., mobile ad hocnetworks or sensor networks.Simulations were conducted to measure the performance of CC-DIF scheme and evaluated the performance in terms of different metrics, such as computation cost, memory consumption, communication overhead and secured message distribution rate in mobile networks. The results show that CC-DIF scheme offers better performance with an improvement of security message distribution rate by 10% and reducing the computation cost for key generations by 20% compared to the state-of-the-art works.

## Supporting information

S1 Appendix Pseudo code for Master Secret Key Generation using Complex Conjugated form. (DOCX)

S2 Appendix Pseudo code for Partial Private Key Extraction using arbitrary bits. (DOCX)

S3 Appendix Pseudo code for signcryption at the sending and unsigncryption at receiving end. (DOCX)

S1 Case File Case examples for Master Secret Key Generation using Complex Conjugated form. (RAR)

S1 Graph File Graph comparison between proposed CC-DIF and existing COOSC, ECC-based MIKEY, CLSC methods. (ZIP)
