# Supplementary material for: Complex Conjugated certificateless-based signcryption with differential integrated factor for secured message communication in mobile network
Source: PLoS One. 2017 Oct 17;12(10):e0186207. doi: 10.1371/journal.pone.0186207 (PMC5645099; doi:10.1371/journal.pone.0186207)
Supplement: S1 Appendix — (DOCX) [file pone.0186207.s001.docx]

/* **Pseudo code for Master Secret Key Generation using Complex Conjugated Form*/**

set val(chan) Channel/WirelessChannel ;# Channel Type

set val(prop) Propagation/TwoRayGround ;# radio-propagation model

set val(netif) Phy/WirelessPhy ;# network interface type

set val(mac) Mac/802_11 ;# MAC type

set val(ifq) Queue/DropTail/PriQueue ;# interface queue type

set val(ll) LL ;# link layer type

set val(ant) Antenna/OmniAntenna ;# antenna model

set val(ifqlen) 50 ;# max packet in ifq

set val(nn) 50 ;# number of mobilenodes

set val(rp) DSR ;# routing protocol

set val(x) 1010

set val(y) 1010

set val(stop) 5.0 ;# simulation time

#-------Event scheduler object creation--------#

set ns [new Simulator]

## Create a trace file and nam file..

set tracefd [open out.tr w]

set namtrace [open out.nam w]

## Trace the nam and trace details from the main simulation..

$ns trace-all $tracefd

$ns namtrace-all-wireless $namtrace $val(x) $val(y)

$ns color 0 purple

## set up topography object..

set topo [new Topography]

$topo load_flatgrid $val(x) $val(y)

set god_ [create-god $val(nn)]

set chan_1_ [new $val(chan)]

# Create node_(0) "attached" to channel #1

# configure node, please note the change below

$ns node-config -adhocRouting $val(rp) \

-llType $val(ll) \

-macType $val(mac) \

-ifqType $val(ifq) \

-ifqLen $val(ifqlen) \

-antType $val(ant) \

-propType $val(prop) \

-phyType $val(netif) \

-topoInstance $topo \

-agentTrace ON \

-routerTrace ON \

-macTrace ON \

-movementTrace ON \

-channel $chan_1_

## Creating node objects...

for {set i 0} {$i < $val(nn) } { incr i } {

set node_($i) [$ns node]

}

for {set i 0} {$i < $val(nn) } {incr i } {

$node_($i) color blue

$ns at 0.0 "$node_($i) color lightgray"

$ns at 0.1 "$node_($i) color black"

}

## Provide initial location of mobilenodes...

for {set i 0} {$i < $val(nn) } { incr i } {

set xx [expr rand()*900]

set yy [expr rand()*900]

$node_($i) set X_ $xx

$node_($i) set Y_ $yy

$node_($i) set Z_ 0.0

}

## Define node initial position in nam...

for {set i 0} {$i < $val(nn)} { incr i } {

# 30 defines the node size for nam..

$ns initial_node_pos $node_($i) 50

}

$ns at 0.0 "$node_(0) setdest 190.0 81.0 11500.0"

$ns at 0.0 "$node_(1) setdest 354.0 830.0 11500.0"

$ns at 0.0 "$node_(2) setdest 642.0 640.0 11500.0"

$ns at 0.0 "$node_(3) setdest 288.0 723.0 11500.0"

$ns at 0.0 "$node_(4) setdest 344.0 311.0 11500.0"

$ns at 0.0 "$node_(5) setdest 712.0 98.0 11500.0"

$ns at 0.0 "$node_(6) setdest 757.0 302.0 11500.0"

$ns at 0.0 "$node_(7) setdest 340.0 187.0 11500.0"

$ns at 0.0 "$node_(8) setdest 540.0 179.0 11500.0"

$ns at 0.0 "$node_(9) setdest 170.5 716.3 11500.0"

$ns at 0.0 "$node_(10) setdest 869.0 94.0 11500.0"

$ns at 0.0 "$node_(11) setdest 499.0 657.0 11500.0"

$ns at 0.0 "$node_(12) setdest 900.0 334.0 11500.0"

$ns at 0.0 "$node_(13) setdest 180.0 215.0 11500.0"

$ns at 0.0 "$node_(14) setdest 558.0 568.0 11500.0"

$ns at 0.0 "$node_(15) setdest 880.0 210.0 11500.0"

$ns at 0.0 "$node_(16) setdest 315.0 458.0 11500.0"

$ns at 0.0 "$node_(17) setdest 46.0 487.0 11500.0"

$ns at 0.0 "$node_(18) setdest 740.0 732.0 11500.0"

$ns at 0.0 "$node_(19) setdest 866.0 604.0 11500.0"

$ns at 0.0 "$node_(20) setdest 507.0 89.0 11500.0"

$ns at 0.0 "$node_(21) setdest 44.0 351.0 11500.0"

$ns at 0.0 "$node_(22) setdest 430.0 750.0 11500.0"

$ns at 0.0 "$node_(23) setdest 760.0 581.0 11500.0"

$ns at 0.0 "$node_(24) setdest 582.0 741.0 11500.0"

$ns at 0.0 "$node_(25) setdest 176.0 618.0 11500.0"

$ns at 0.0 "$node_(26) setdest 34.000000 604.889404 11500.0"

$ns at 0.0 "$node_(27) setdest 64.0 722.0 11500.0"

$ns at 0.0 "$node_(28) setdest 215.0 829.0 11500.0"

$ns at 0.0 "$node_(29) setdest 550.0 837.0 11500.0"

$ns at 0.0 "$node_(30) setdest 530.0 306.0 11500.0"

$ns at 0.0 "$node_(31) setdest 364.0 99.0 11500.0"

$ns at 0.0 "$node_(32) setdest 622.0 453.0 11500.0"

$ns at 0.0 "$node_(33) setdest 639.0 336.0 11500.0"

$ns at 0.0 "$node_(34) setdest 840.7 730.1 11500.0"

$ns at 0.0 "$node_(35) setdest 84.0 810.0 11500.0"

$ns at 0.0 "$node_(36) setdest 785.0 204.0 11500.0"

$ns at 0.0 "$node_(37) setdest 275.028870 614.596130 11500.0"

$ns at 0.0 "$node_(38) setdest 75.0 111.0 11500.0"

$ns at 0.0 "$node_(39) setdest 177.0 473.0 11500.0"

$ns at 0.0 "$node_(40) setdest 752.0 454.0 11500.0"

$ns at 0.0 "$node_(41) setdest 52.0 232.0 11500.0"

$ns at 0.0 "$node_(42) setdest 203.0 355.0 11500.0"

$ns at 0.0 "$node_(43) setdest 700.0 820.0 11500.0"

$ns at 0.0 "$node_(44) setdest 890.0 460.0 11500.0"

$ns at 0.0 "$node_(45) setdest 476.0 471.0 11500.0"

$ns at 0.0 "$node_(46) setdest 427.0 360.0 11500.0"

$ns at 0.0 "$node_(47) setdest 390.0 588.0 11500.0"

$ns at 0.0 "$node_(48) setdest 815.0 830.4 11500.0"

$ns at 0.0 "$node_(49) setdest 661.0 220.0 11500.0"

$ns at 0.8 "$node_(17) label Sender"

$ns at 0.8 "$node_(44) label Receiver"

$ns at 1.0 "$node_(17) color blue"

$ns at 1.0 "$node_(44) color blue"

$ns at 1.2 "$node_(17) add-mark m1 brown hexagon"

$ns at 1.2 "$node_(44) add-mark m2 brown hexagon"

$ns at 2.02 "$node_(26) color pink"

$ns at 2.04 "$node_(27) color pink"

$ns at 2.06 "$node_(9) color pink"

$ns at 2.08 "$node_(3) color pink"

$ns at 2.1 "$node_(22) color pink"

$ns at 2.12 "$node_(24) color pink"

$ns at 2.14 "$node_(18) color pink"

$ns at 2.16 "$node_(34) color pink"

$ns at 2.18 "$node_(19) color pink"

$ns at 2.22 "$node_(39) color pink"

$ns at 2.24 "$node_(16) color pink"

$ns at 2.26 "$node_(45) color pink"

$ns at 2.28 "$node_(32) color pink"

$ns at 2.3 "$node_(40) color pink"

$ns at 2.34 "$node_(21) color pink"

$ns at 2.36 "$node_(41) color pink"

$ns at 2.38 "$node_(13) color pink"

$ns at 2.4 "$node_(7) color pink"

$ns at 2.42 "$node_(8) color pink"

$ns at 2.44 "$node_(49) color pink"

$ns at 2.46 "$node_(36) color pink"

$ns at 2.48 "$node_(15) color pink"

$ns at 2.5 "$node_(12) color pink"

$ns at 2.6 "$node_(26) label Master_Secret_Key"

$ns at 2.8 "$node_(39) label MSK"

$ns at 2.92 "$node_(21) label MSK"

$ns at 2.6 "$node_(26) color brown"

$ns at 2.8 "$node_(39) color brown"

$ns at 2.92 "$node_(21) color brown"

$ns at 2.78 "$node_(19) label rec_MSK"

$ns at 2.9 "$node_(40) label rec_MSK"

$ns at 3.1 "$node_(12) label rec_MSK"

$ns at 2.78 "$node_(19) color brown"

$ns at 2.9 "$node_(40) color brown"

$ns at 3.1 "$node_(12) color brown"

#*********************************************Complex Conjugated Master Secret Key Generation*************************************************

set Users unknown

#data packets

proc ComplexConjugatedMasterSecretKeyGeneration{value(DP) } {

Begin

{

for{set $ID 1}{$ID < n} {$ID incr}

{

Generate System Parameter->(MPK,ms,cs)

Generate Complex Conjugate

}

End for

}

End

}

#***************************************************************************************************************************

set tcp5 [new Agent/TCP]

set sink6 [new Agent/TCPSink]

$ns attach-agent $node_(17) $tcp5

$ns attach-agent $node_(26) $sink6

$ns connect $tcp5 $sink6

set ftp5 [new Application/FTP]

$ftp5 attach-agent $tcp5

$ns at 2.0 "$ftp5 start"

$ns at 2.02 "$ftp5 stop"

set tcp5 [new Agent/TCP]

set sink6 [new Agent/TCPSink]

$ns attach-agent $node_(26) $tcp5

$ns attach-agent $node_(27) $sink6

$ns connect $tcp5 $sink6

set ftp5 [new Application/FTP]

$ftp5 attach-agent $tcp5

$ns at 2.02 "$ftp5 start"

$ns at 2.04 "$ftp5 stop"

set tcp5 [new Agent/TCP]

set sink6 [new Agent/TCPSink]

$ns attach-agent $node_(27) $tcp5

$ns attach-agent $node_(9) $sink6

$ns connect $tcp5 $sink6

set ftp5 [new Application/FTP]

$ftp5 attach-agent $tcp5

$ns at 2.04 "$ftp5 start"

$ns at 2.06 "$ftp5 stop"

set tcp5 [new Agent/TCP]

set sink6 [new Agent/TCPSink]

$ns attach-agent $node_(9) $tcp5

$ns attach-agent $node_(3) $sink6

$ns connect $tcp5 $sink6

set ftp5 [new Application/FTP]

$ftp5 attach-agent $tcp5

$ns at 2.06 "$ftp5 start"

$ns at 2.08 "$ftp5 stop"

set tcp5 [new Agent/TCP]

set sink6 [new Agent/TCPSink]

$ns attach-agent $node_(3) $tcp5

$ns attach-agent $node_(22) $sink6

$ns connect $tcp5 $sink6

set ftp5 [new Application/FTP]

$ftp5 attach-agent $tcp5

$ns at 2.08 "$ftp5 start"

$ns at 2.1 "$ftp5 stop"

set tcp5 [new Agent/TCP]

set sink6 [new Agent/TCPSink]

$ns attach-agent $node_(22) $tcp5

$ns attach-agent $node_(24) $sink6

$ns connect $tcp5 $sink6

set ftp5 [new Application/FTP]

$ftp5 attach-agent $tcp5

$ns at 2.1 "$ftp5 start"

$ns at 2.12 "$ftp5 stop"

set tcp5 [new Agent/TCP]

set sink6 [new Agent/TCPSink]

$ns attach-agent $node_(24) $tcp5

$ns attach-agent $node_(18) $sink6

$ns connect $tcp5 $sink6

set ftp5 [new Application/FTP]

$ftp5 attach-agent $tcp5

$ns at 2.12 "$ftp5 start"

$ns at 2.14 "$ftp5 stop"

set tcp5 [new Agent/TCP]

set sink6 [new Agent/TCPSink]

$ns attach-agent $node_(18) $tcp5

$ns attach-agent $node_(34) $sink6

$ns connect $tcp5 $sink6

set ftp5 [new Application/FTP]

$ftp5 attach-agent $tcp5

$ns at 2.14 "$ftp5 start"

$ns at 2.16 "$ftp5 stop"

set tcp5 [new Agent/TCP]

set sink6 [new Agent/TCPSink]

$ns attach-agent $node_(34) $tcp5

$ns attach-agent $node_(19) $sink6

$ns connect $tcp5 $sink6

set ftp5 [new Application/FTP]

$ftp5 attach-agent $tcp5

$ns at 2.16 "$ftp5 start"

$ns at 2.18 "$ftp5 stop"

set tcp5 [new Agent/TCP]

set sink6 [new Agent/TCPSink]

$ns attach-agent $node_(19) $tcp5

$ns attach-agent $node_(44) $sink6

$ns connect $tcp5 $sink6

set ftp5 [new Application/FTP]

$ftp5 attach-agent $tcp5

$ns at 2.18 "$ftp5 start"

$ns at 2.2 "$ftp5 stop"

set tcp5 [new Agent/TCP]

set sink6 [new Agent/TCPSink]

$ns attach-agent $node_(17) $tcp5

$ns attach-agent $node_(39) $sink6

$ns connect $tcp5 $sink6

set ftp5 [new Application/FTP]

$ftp5 attach-agent $tcp5

$ns at 2.2 "$ftp5 start"

$ns at 2.22 "$ftp5 stop"

set tcp5 [new Agent/TCP]

set sink6 [new Agent/TCPSink]

$ns attach-agent $node_(39) $tcp5

$ns attach-agent $node_(16) $sink6

$ns connect $tcp5 $sink6

set ftp5 [new Application/FTP]

$ftp5 attach-agent $tcp5

$ns at 2.22 "$ftp5 start"

$ns at 2.24 "$ftp5 stop"

set tcp5 [new Agent/TCP]

set sink6 [new Agent/TCPSink]

$ns attach-agent $node_(16) $tcp5

$ns attach-agent $node_(45) $sink6

$ns connect $tcp5 $sink6

set ftp5 [new Application/FTP]

$ftp5 attach-agent $tcp5

$ns at 2.24 "$ftp5 start"

$ns at 2.26 "$ftp5 stop"

set tcp5 [new Agent/TCP]

set sink6 [new Agent/TCPSink]

$ns attach-agent $node_(45) $tcp5

$ns attach-agent $node_(32) $sink6

$ns connect $tcp5 $sink6

set ftp5 [new Application/FTP]

$ftp5 attach-agent $tcp5

$ns at 2.26 "$ftp5 start"

$ns at 2.28 "$ftp5 stop"

set tcp5 [new Agent/TCP]

set sink6 [new Agent/TCPSink]

$ns attach-agent $node_(32) $tcp5

$ns attach-agent $node_(40) $sink6

$ns connect $tcp5 $sink6

set ftp5 [new Application/FTP]

$ftp5 attach-agent $tcp5

$ns at 2.28 "$ftp5 start"

$ns at 2.3 "$ftp5 stop"

set tcp5 [new Agent/TCP]

set sink6 [new Agent/TCPSink]

$ns attach-agent $node_(40) $tcp5

$ns attach-agent $node_(44) $sink6

$ns connect $tcp5 $sink6

set ftp5 [new Application/FTP]

$ftp5 attach-agent $tcp5

$ns at 2.3 "$ftp5 start"

$ns at 2.32 "$ftp5 stop"

set tcp5 [new Agent/TCP]

set sink6 [new Agent/TCPSink]

$ns attach-agent $node_(17) $tcp5

$ns attach-agent $node_(21) $sink6

$ns connect $tcp5 $sink6

set ftp5 [new Application/FTP]

$ftp5 attach-agent $tcp5

$ns at 2.32 "$ftp5 start"

$ns at 2.34 "$ftp5 stop"

set tcp5 [new Agent/TCP]

set sink6 [new Agent/TCPSink]

$ns attach-agent $node_(21) $tcp5

$ns attach-agent $node_(41) $sink6

$ns connect $tcp5 $sink6

set ftp5 [new Application/FTP]

$ftp5 attach-agent $tcp5

$ns at 2.34 "$ftp5 start"

$ns at 2.36 "$ftp5 stop"

set tcp5 [new Agent/TCP]

set sink6 [new Agent/TCPSink]

$ns attach-agent $node_(41) $tcp5

$ns attach-agent $node_(13) $sink6

$ns connect $tcp5 $sink6

set ftp5 [new Application/FTP]

$ftp5 attach-agent $tcp5

$ns at 2.36 "$ftp5 start"

$ns at 2.38 "$ftp5 stop"

set tcp5 [new Agent/TCP]

set sink6 [new Agent/TCPSink]

$ns attach-agent $node_(13) $tcp5

$ns attach-agent $node_(7) $sink6

$ns connect $tcp5 $sink6

set ftp5 [new Application/FTP]

$ftp5 attach-agent $tcp5

$ns at 2.38 "$ftp5 start"

$ns at 2.4 "$ftp5 stop"

set tcp5 [new Agent/TCP]

set sink6 [new Agent/TCPSink]

$ns attach-agent $node_(7) $tcp5

$ns attach-agent $node_(8) $sink6

$ns connect $tcp5 $sink6

set ftp5 [new Application/FTP]

$ftp5 attach-agent $tcp5

$ns at 2.4 "$ftp5 start"

$ns at 2.42 "$ftp5 stop"

set tcp5 [new Agent/TCP]

set sink6 [new Agent/TCPSink]

$ns attach-agent $node_(8) $tcp5

$ns attach-agent $node_(49) $sink6

$ns connect $tcp5 $sink6

set ftp5 [new Application/FTP]

$ftp5 attach-agent $tcp5

$ns at 2.42 "$ftp5 start"

$ns at 2.44 "$ftp5 stop"

set tcp5 [new Agent/TCP]

set sink6 [new Agent/TCPSink]

$ns attach-agent $node_(49) $tcp5

$ns attach-agent $node_(36) $sink6

$ns connect $tcp5 $sink6

set ftp5 [new Application/FTP]

$ftp5 attach-agent $tcp5

$ns at 2.44 "$ftp5 start"

$ns at 2.46 "$ftp5 stop"

set tcp5 [new Agent/TCP]

set sink6 [new Agent/TCPSink]

$ns attach-agent $node_(36) $tcp5

$ns attach-agent $node_(15) $sink6

$ns connect $tcp5 $sink6

set ftp5 [new Application/FTP]

$ftp5 attach-agent $tcp5

$ns at 2.46 "$ftp5 start"

$ns at 2.48 "$ftp5 stop"

set tcp5 [new Agent/TCP]

set sink6 [new Agent/TCPSink]

$ns attach-agent $node_(15) $tcp5

$ns attach-agent $node_(12) $sink6

$ns connect $tcp5 $sink6

set ftp5 [new Application/FTP]

$ftp5 attach-agent $tcp5

$ns at 2.48 "$ftp5 start"

$ns at 2.5 "$ftp5 stop"

set tcp5 [new Agent/TCP]

set sink6 [new Agent/TCPSink]

$ns attach-agent $node_(12) $tcp5

$ns attach-agent $node_(44) $sink6

$ns connect $tcp5 $sink6

set ftp5 [new Application/FTP]

$ftp5 attach-agent $tcp5

$ns at 2.5 "$ftp5 start"

$ns at 2.52 "$ftp5 stop"

set tcp5 [new Agent/TCP]

set sink6 [new Agent/TCPSink]

$ns attach-agent $node_(17) $tcp5

$ns attach-agent $node_(26) $sink6

$ns connect $tcp5 $sink6

set ftp5 [new Application/FTP]

$ftp5 attach-agent $tcp5

$ns at 2.6 "$ftp5 start"

$ns at 2.62 "$ftp5 stop"

set tcp5 [new Agent/TCP]

set sink6 [new Agent/TCPSink]

$ns attach-agent $node_(26) $tcp5

$ns attach-agent $node_(27) $sink6

$ns connect $tcp5 $sink6

set ftp5 [new Application/FTP]

$ftp5 attach-agent $tcp5

$ns at 2.62 "$ftp5 start"

$ns at 2.64 "$ftp5 stop"

set tcp5 [new Agent/TCP]

set sink6 [new Agent/TCPSink]

$ns attach-agent $node_(27) $tcp5

$ns attach-agent $node_(9) $sink6

$ns connect $tcp5 $sink6

set ftp5 [new Application/FTP]

$ftp5 attach-agent $tcp5

$ns at 2.64 "$ftp5 start"

$ns at 2.66 "$ftp5 stop"

set tcp5 [new Agent/TCP]

set sink6 [new Agent/TCPSink]

$ns attach-agent $node_(9) $tcp5

$ns attach-agent $node_(3) $sink6

$ns connect $tcp5 $sink6

set ftp5 [new Application/FTP]

$ftp5 attach-agent $tcp5

$ns at 2.66 "$ftp5 start"

$ns at 2.68 "$ftp5 stop"

set tcp5 [new Agent/TCP]

set sink6 [new Agent/TCPSink]

$ns attach-agent $node_(3) $tcp5

$ns attach-agent $node_(22) $sink6

$ns connect $tcp5 $sink6

set ftp5 [new Application/FTP]

$ftp5 attach-agent $tcp5

$ns at 2.68 "$ftp5 start"

$ns at 2.7 "$ftp5 stop"

set tcp5 [new Agent/TCP]

set sink6 [new Agent/TCPSink]

$ns attach-agent $node_(22) $tcp5

$ns attach-agent $node_(24) $sink6

$ns connect $tcp5 $sink6

set ftp5 [new Application/FTP]

$ftp5 attach-agent $tcp5

$ns at 2.7 "$ftp5 start"

$ns at 2.72 "$ftp5 stop"

set tcp5 [new Agent/TCP]

set sink6 [new Agent/TCPSink]

$ns attach-agent $node_(24) $tcp5

$ns attach-agent $node_(18) $sink6

$ns connect $tcp5 $sink6

set ftp5 [new Application/FTP]

$ftp5 attach-agent $tcp5

$ns at 2.72 "$ftp5 start"

$ns at 2.74 "$ftp5 stop"

set tcp5 [new Agent/TCP]

set sink6 [new Agent/TCPSink]

$ns attach-agent $node_(18) $tcp5

$ns attach-agent $node_(34) $sink6

$ns connect $tcp5 $sink6

set ftp5 [new Application/FTP]

$ftp5 attach-agent $tcp5

$ns at 2.74 "$ftp5 start"

$ns at 2.76 "$ftp5 stop"

set tcp5 [new Agent/TCP]

set sink6 [new Agent/TCPSink]

$ns attach-agent $node_(34) $tcp5

$ns attach-agent $node_(19) $sink6

$ns connect $tcp5 $sink6

set ftp5 [new Application/FTP]

$ftp5 attach-agent $tcp5

$ns at 2.76 "$ftp5 start"

$ns at 2.78 "$ftp5 stop"

set tcp5 [new Agent/TCP]

set sink6 [new Agent/TCPSink]

$ns attach-agent $node_(19) $tcp5

$ns attach-agent $node_(44) $sink6

$ns connect $tcp5 $sink6

set ftp5 [new Application/FTP]

$ftp5 attach-agent $tcp5

$ns at 2.78 "$ftp5 start"

$ns at 2.8 "$ftp5 stop"

set tcp5 [new Agent/TCP]

set sink6 [new Agent/TCPSink]

$ns attach-agent $node_(17) $tcp5

$ns attach-agent $node_(39) $sink6

$ns connect $tcp5 $sink6

set ftp5 [new Application/FTP]

$ftp5 attach-agent $tcp5

$ns at 2.8 "$ftp5 start"

$ns at 2.82 "$ftp5 stop"

set tcp5 [new Agent/TCP]

set sink6 [new Agent/TCPSink]

$ns attach-agent $node_(39) $tcp5

$ns attach-agent $node_(16) $sink6

$ns connect $tcp5 $sink6

set ftp5 [new Application/FTP]

$ftp5 attach-agent $tcp5

$ns at 2.82 "$ftp5 start"

$ns at 2.84 "$ftp5 stop"

set tcp5 [new Agent/TCP]

set sink6 [new Agent/TCPSink]

$ns attach-agent $node_(16) $tcp5

$ns attach-agent $node_(45) $sink6

$ns connect $tcp5 $sink6

set ftp5 [new Application/FTP]

$ftp5 attach-agent $tcp5

$ns at 2.84 "$ftp5 start"

$ns at 2.86 "$ftp5 stop"

set tcp5 [new Agent/TCP]

set sink6 [new Agent/TCPSink]

$ns attach-agent $node_(45) $tcp5

$ns attach-agent $node_(32) $sink6

$ns connect $tcp5 $sink6

set ftp5 [new Application/FTP]

$ftp5 attach-agent $tcp5

$ns at 2.86 "$ftp5 start"

$ns at 2.88 "$ftp5 stop"

set tcp5 [new Agent/TCP]

set sink6 [new Agent/TCPSink]

$ns attach-agent $node_(32) $tcp5

$ns attach-agent $node_(40) $sink6

$ns connect $tcp5 $sink6

set ftp5 [new Application/FTP]

$ftp5 attach-agent $tcp5

$ns at 2.88 "$ftp5 start"

$ns at 2.9 "$ftp5 stop"

set tcp5 [new Agent/TCP]

set sink6 [new Agent/TCPSink]

$ns attach-agent $node_(40) $tcp5

$ns attach-agent $node_(44) $sink6

$ns connect $tcp5 $sink6

set ftp5 [new Application/FTP]

$ftp5 attach-agent $tcp5

$ns at 2.9 "$ftp5 start"

$ns at 2.92 "$ftp5 stop"

set tcp5 [new Agent/TCP]

set sink6 [new Agent/TCPSink]

$ns attach-agent $node_(17) $tcp5

$ns attach-agent $node_(21) $sink6

$ns connect $tcp5 $sink6

set ftp5 [new Application/FTP]

$ftp5 attach-agent $tcp5

$ns at 2.92 "$ftp5 start"

$ns at 2.94 "$ftp5 stop"

set tcp5 [new Agent/TCP]

set sink6 [new Agent/TCPSink]

$ns attach-agent $node_(21) $tcp5

$ns attach-agent $node_(41) $sink6

$ns connect $tcp5 $sink6

set ftp5 [new Application/FTP]

$ftp5 attach-agent $tcp5

$ns at 2.94 "$ftp5 start"

$ns at 2.96 "$ftp5 stop"

set tcp5 [new Agent/TCP]

set sink6 [new Agent/TCPSink]

$ns attach-agent $node_(41) $tcp5

$ns attach-agent $node_(13) $sink6

$ns connect $tcp5 $sink6

set ftp5 [new Application/FTP]

$ftp5 attach-agent $tcp5

$ns at 2.96 "$ftp5 start"

$ns at 2.98 "$ftp5 stop"

set tcp5 [new Agent/TCP]

set sink6 [new Agent/TCPSink]

$ns attach-agent $node_(13) $tcp5

$ns attach-agent $node_(7) $sink6

$ns connect $tcp5 $sink6

set ftp5 [new Application/FTP]

$ftp5 attach-agent $tcp5

$ns at 2.98 "$ftp5 start"

$ns at 3.0 "$ftp5 stop"

set tcp5 [new Agent/TCP]

set sink6 [new Agent/TCPSink]

$ns attach-agent $node_(7) $tcp5

$ns attach-agent $node_(8) $sink6

$ns connect $tcp5 $sink6

set ftp5 [new Application/FTP]

$ftp5 attach-agent $tcp5

$ns at 3.0 "$ftp5 start"

$ns at 3.02 "$ftp5 stop"

set tcp5 [new Agent/TCP]

set sink6 [new Agent/TCPSink]

$ns attach-agent $node_(8) $tcp5

$ns attach-agent $node_(49) $sink6

$ns connect $tcp5 $sink6

set ftp5 [new Application/FTP]

$ftp5 attach-agent $tcp5

$ns at 3.02 "$ftp5 start"

$ns at 3.04 "$ftp5 stop"

set tcp5 [new Agent/TCP]

set sink6 [new Agent/TCPSink]

$ns attach-agent $node_(49) $tcp5

$ns attach-agent $node_(36) $sink6

$ns connect $tcp5 $sink6

set ftp5 [new Application/FTP]

$ftp5 attach-agent $tcp5

$ns at 3.04 "$ftp5 start"

$ns at 3.06 "$ftp5 stop"

set tcp5 [new Agent/TCP]

set sink6 [new Agent/TCPSink]

$ns attach-agent $node_(36) $tcp5

$ns attach-agent $node_(15) $sink6

$ns connect $tcp5 $sink6

set ftp5 [new Application/FTP]

$ftp5 attach-agent $tcp5

$ns at 3.06 "$ftp5 start"

$ns at 3.08 "$ftp5 stop"

set tcp5 [new Agent/TCP]

set sink6 [new Agent/TCPSink]

$ns attach-agent $node_(15) $tcp5

$ns attach-agent $node_(12) $sink6

$ns connect $tcp5 $sink6

set ftp5 [new Application/FTP]

$ftp5 attach-agent $tcp5

$ns at 3.08 "$ftp5 start"

$ns at 3.1 "$ftp5 stop"

set tcp5 [new Agent/TCP]

set sink6 [new Agent/TCPSink]

$ns attach-agent $node_(12) $tcp5

$ns attach-agent $node_(44) $sink6

$ns connect $tcp5 $sink6

set ftp5 [new Application/FTP]

$ftp5 attach-agent $tcp5

$ns at 3.1 "$ftp5 start"

$ns at 3.12 "$ftp5 stop"

#***************************************************************************************************************************

$ns at 0.2 "$ns trace-annotate \"Master Secret Key Generation using Complex Conjugated form- Module1\""

$ns at 0.7 "$ns trace-annotate \"The setup phase in CC-DIF scheme provides security parameters as input and obtains the Master Secret Key using Complex Conjugate form.\""

$ns at 0.9 "$ns trace-annotate \"Master Secret Key is obtained using the complex conjugate form of the security parameter and the security parameter respectively at time.\""

$ns at 1.2 "$ns trace-annotate \"Security parameters as input, for all users, master secret key and system parameters are generated as output.\""

$ns at 1.5 "$ns trace-annotate \"linearly independent terms are obtained using and the secured message distribution rate is said to be improved.\""

$ns at 2.6 "$ns trace-annotate \"Traverse through the path 17-26-27-9-3-22-24-18-34-19-44\""

$ns at 2.8 "$ns trace-annotate \"Traverse through the path 17-39-16-45-32-40-44\""

$ns at 2.92 "$ns trace-annotate \"Traverse through the path 17-21-41-13-7-8-49-36-15-12-44\""

#***************************************************************************************************************************

## stop procedure..

$ns at $val(stop) "stop"

proc stop {} {

global ns tracefd namtrace

$ns flush-trace

close $tracefd

close $namtrace

puts "running nam..."

exec nam out &

exit 0

}

$ns run
