# Supplementary figures and images for: Complex Conjugated certificateless-based signcryption with differential integrated factor for secured message communication in mobile network
Source: PLoS One. 2017 Oct 17;12(10):e0186207. doi: 10.1371/journal.pone.0186207 (PMC5645099; doi:10.1371/journal.pone.0186207)

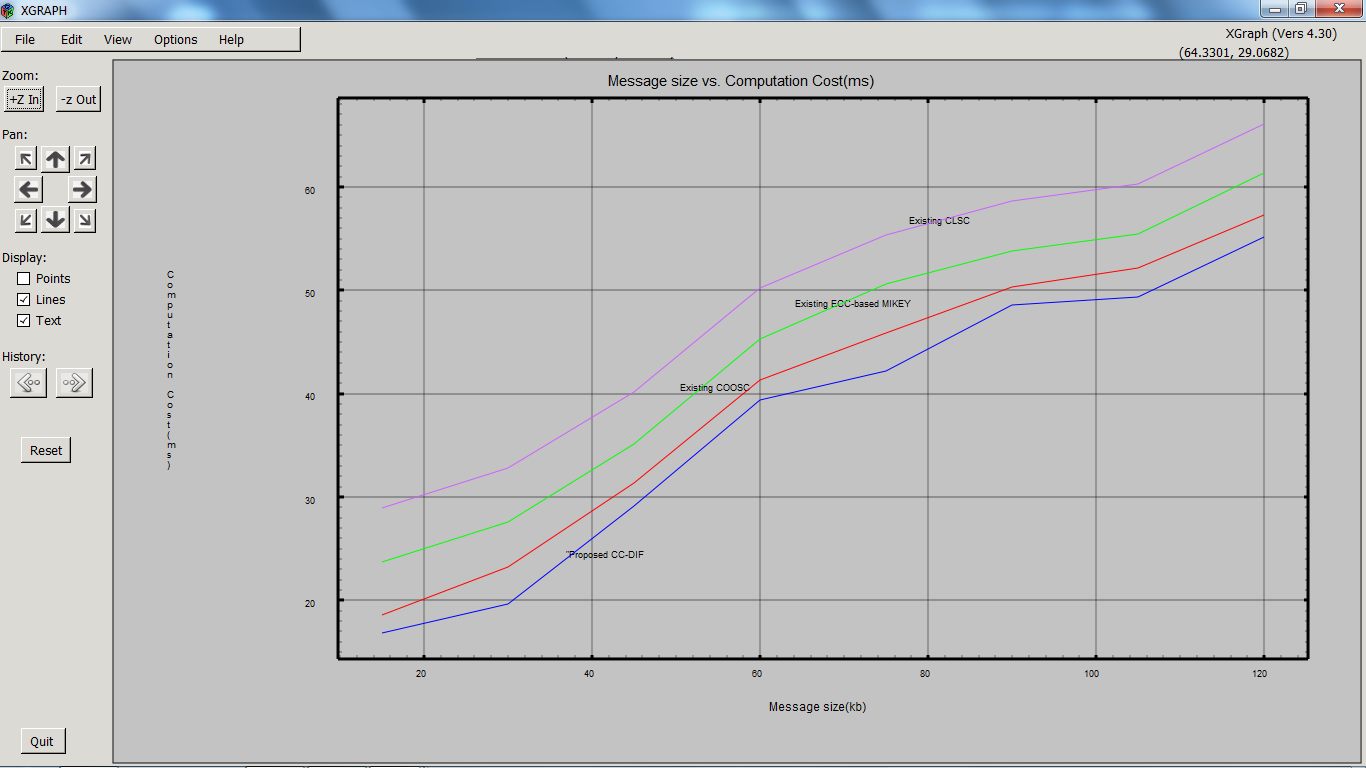

Supplement: S1 Graph File — (ZIP) [file pone.0186207.s005.zip › graph/g1.png]

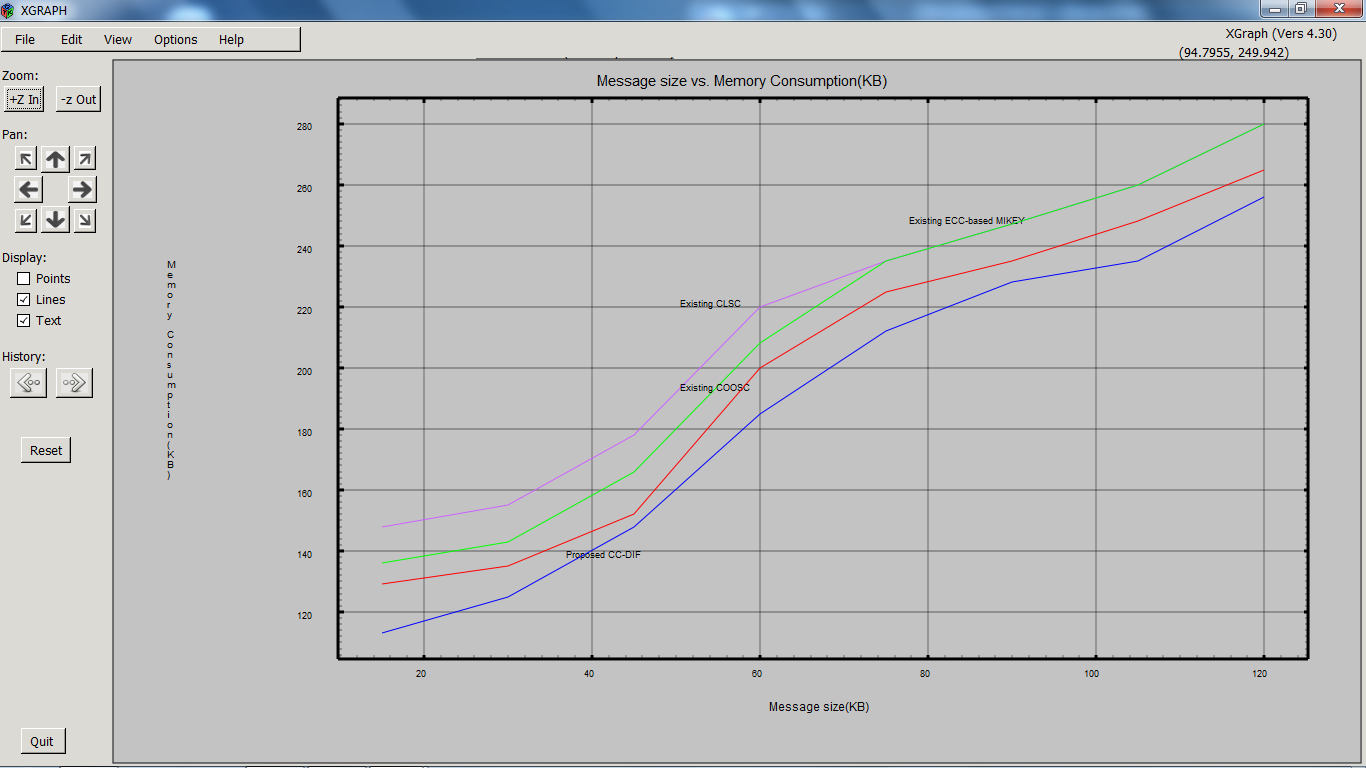

Supplement: S1 Graph File — (ZIP) [file pone.0186207.s005.zip › graph/g2.png]

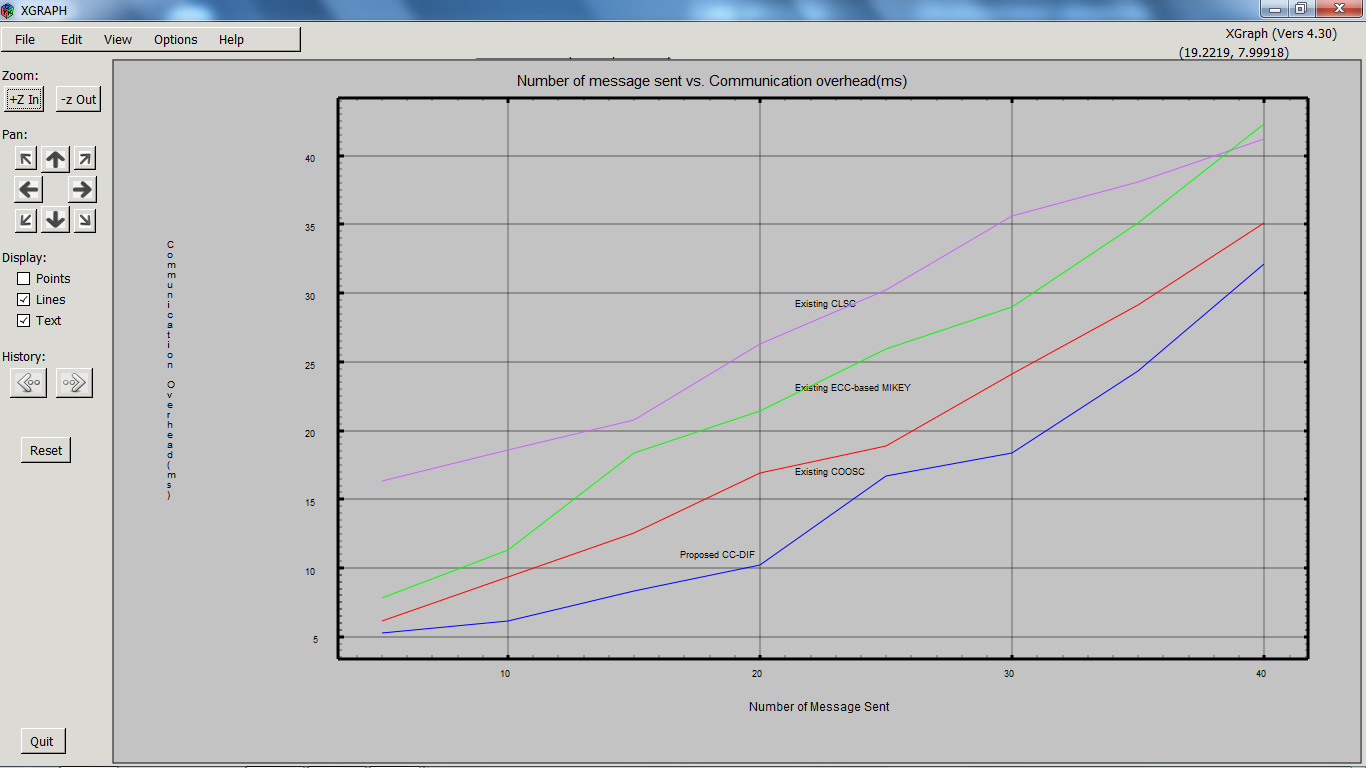

Supplement: S1 Graph File — (ZIP) [file pone.0186207.s005.zip › graph/g3.png]

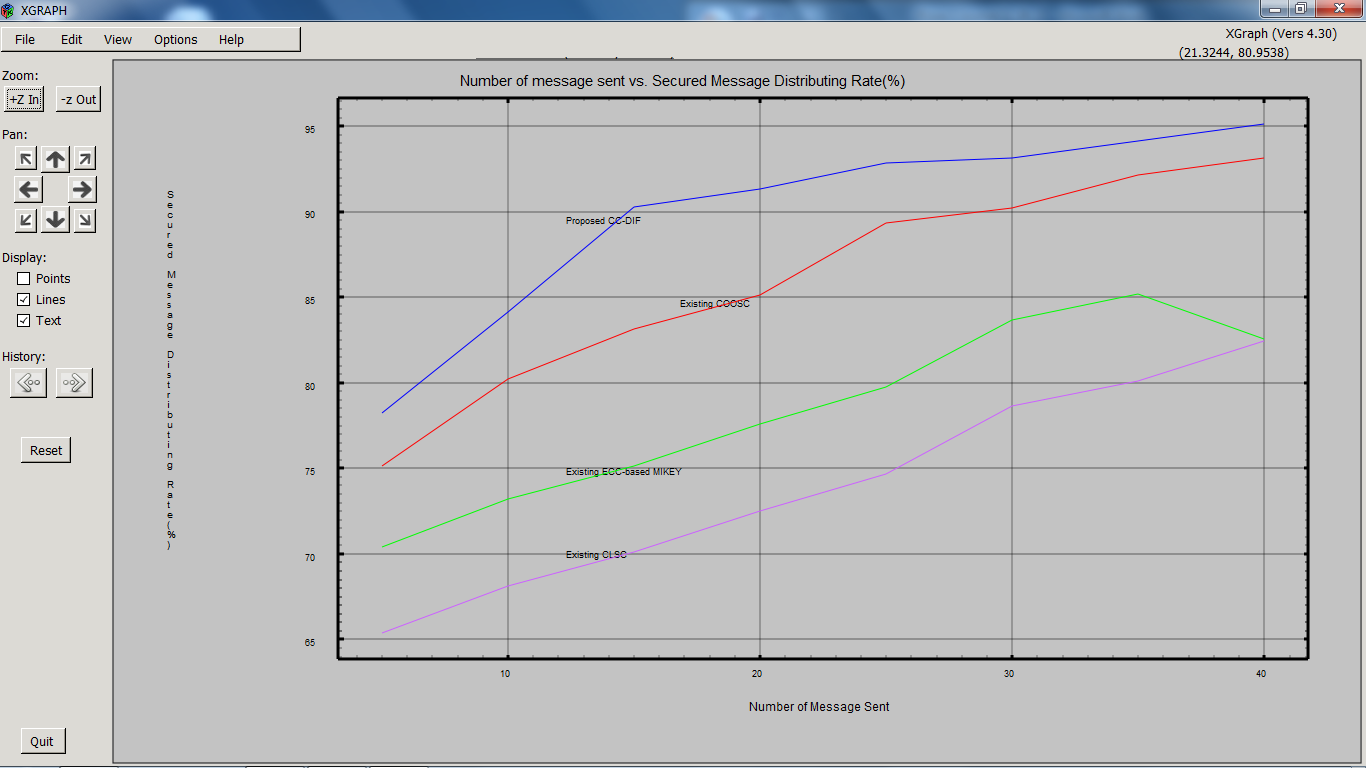

Supplement: S1 Graph File — (ZIP) [file pone.0186207.s005.zip › graph/g4.png]
